# The related factors of sleep benefit in Parkinson’s disease: A systematic review and meta-analysis

**DOI:** 10.1371/journal.pone.0212951

**Published:** 2019-03-11

**Authors:** Zhong Rui, Chen Qingling, Zhang Xinyue, Zhang Xin, Lin Weihong

**Affiliations:** 1 Department of Neurology, The First Hospital of Jilin University, Chang Chun, Ji Lin Provence, China; 2 Digestive Department, The First Hospital of Jilin University, Chang Chun, Ji Lin Provence, China; IRCCS Istituto Delle Scienze Neurologiche di Bologna, ITALY

## Abstract

**Background:**

Sleep benefit (SB) refers to the poorly understood phenomenon in Parkinson’s disease (PD) in which patients wake up in the morning with improved motor function. Although previous studies have suggested that several related factors are associated with SB, this relationship remains controversial.

**Objective:**

This systematic review and meta-analysis aimed to identify the possible related factors of SB in PD.

**Methods:**

We searched PubMed, EMBASE and WanFang databases to collect eligible articles. We calculated pooled estimates of odds ratios (ORs) or the mean deviation (MD) with 95% confidence intervals.

**Results:**

We found 3 related factors associated with SB: the duration of PD (MD 1.22, 95% CI: 0.21–2.23), sleep efficiency (MD -4.48,95% CI: -7.24- -2.44), and on-state MDS-UPDRS-Ⅲ total score (MD 3.05, 95% CI: 0.53–5.57).

**Conclusion:**

PD patients with SB are more likely to have a long duration of PD, a low sleep efficiency and a high MDS-UPDRS-Ⅲ total score. Our work helps obtain a better understanding of sleep SB in PD and its underlying mechanisms. More studies need to be conducted to evaluate the associations between clinical factors in PD and the SB phenomenon.

## Introduction

Parkinson’s disease (PD) is a chronic, progressive, disabling neurodegenerative disorder that begins in mid to late life and is characterized by motor impairment, autonomic dysfunction, and, in many patients, psychological and cognitive changes[1, 2]. Sleep disorders are common among Parkinson’s disease(PD) patients and included insomnia, excessive sleeping, and restless legs syndrome, which can reduce the quality of life in PD patients[3, 4]. Furthermore, limited treatment options are available [5]. However, there are also reports of PD patients experiencing a beneficial effect of sleep. Upon waking in the morning, some patients describe a good mobility, as if they are in an ‘on’ state induced by medication, contrary to what would be expected after a night without medication[6]. PD patients with sleep benefit(SB) even feel “this is my best time, as if I had no disease or it were much milder”, when they wake up in the morning before taking any medication. Some patients are even able to delay or decrease the dose of morning medication due to sleep benefit(SB)[7]. Therefore, sleep benefit is the experience of a temporary decrease in PD symptoms upon awakening after a period of sleep (night or daytime) and before drug intake; the patient is feeling as good as “on” (or better); thus, it is a subjective measure. Sleep is believed to improve the extrapyramidal motor functions of these patients[8]. However, the subjective versus objective SB in PD is controversial, and several previous studies revealed a significant discrepancy between subjective functional and objective motor improvement. A study by Lee et al concluded that most PD patients experience subjective SB with no measurable motor improvement[9].

A broad range of possible demographic and clinical determinants, such as age, gender, age of PD onset, disease duration, medication use, etc, have been assessed as possible related factors for SB in patients with PD; however, different outcomes have been reported in the different studies. A study by W. Lee et al[9] suggested that no clinical characteristics differentiated patients with and without SB. However, age, age at PD onset, time of PD from diagnosis, time on treatment, LEDD (L-dopa equivalent daily dose), total sleep duration and sleep efficiency were all considered as related factors for SB in PD patients in another study[10]. We conducted this systematic review and meta-analysis to identify the possible related factors of SB in PD, with the aim of obtaining a better understanding of SB in PD and its underlying mechanisms.

## Materials and methods

### Literature search

We systematically searched PubMed and EMBASE to collect eligible articles using the key search terms”sleep benefit” AND “Parkinson’s disease”, Then, we searched the WanFang database for eligible studies with the terms” shuimianhuoyi” AND “pajinsenbing”.Studies were published from database inception to Oct 10, 2018. We also reviewed the reference lists of the included studies to identify additional articles. We restricted our search to clinical observational studies. Only Chinese and English articles were involved.

### Study selection and quality assessment

The studies included in this meta-analysis met the following inclusion criteria: (1) clinical observational study design; (2) inclusion of a comparison between PD patients with SB and PD patients without SB; (3) reporting of possible demographic and clinical determinants, such as age, gender, age of PD onset, disease duration, medication use, etc, as outcomes; (4) reporting of the number of individuals in each group and providing of sufficient data for the meta-analysis; and (5) PD was diagnosed based on standard criteria, SB on motor function were identified by subjective phenomenon reported by PD patients or objective measurements. The exclusion criteria were as follows: (1) duplicates; (2) the number of individuals in each group and sufficient data were not reported; and, (3) case reports, letters, reviews and conference abstracts. We first screened titles and abstracts and excluded studies that obviously did not fulfill the inclusion criteria. Studies that provisionally met the eligibility criteria were assessed for eligibility by examining the full text. Two reviewers (Zhong and Chen) independently checked the articles, and resolved disagreements by discussion.

The quality of the included studies was subjectively graded using the Newcastle-Ottawa scales for cross-sectional studies. A study was considered high quality when the total score was at least 6. Two reviewers independently evaluated the quality of the eligible conventional studies, and resolved disagreements by discussion.

### Date abstraction

We abstracted the base characteristics of these included studies, which included the authors name, publication date, country, race, diagnostic criteria and the number of individuals in each group. We also abstracted detailed information, such as age, gender, age of PD onset, disease duration, medication use, etc. Two investigators (Zhong and Chen) independently abstracted data from eligible articles, and any discrepant judgment were resolved by joint discussion.

### Statistical analysis

Heterogeneity was assessed by the P value of the X^2 and I^2 statistics, and heterogeneity was significant if the I^2 statistic was greater than 50% or the P value was less than 0.5. A meta-analysis was conducted if the related factor was reported in at least 2 studies. The detailed information as reported in the studies for each possible factor was used. Odds ratios (ORs) or mean deviations (MDs) with 95% confidence intervals were used as a measure of the association of SB in PD with the possible factors, and the results were presented as forest plots, which included the contribution of each study (weight) to the overall effect. We used randomized effects models to pool the results for significant heterogeneity in our meta-analysis; otherwise, a fixed effects model was applied. The Review Manager Software Package (RevMan 5.3) was used for this meta-analysis.

## Results

### Study search

**Fig 1:**
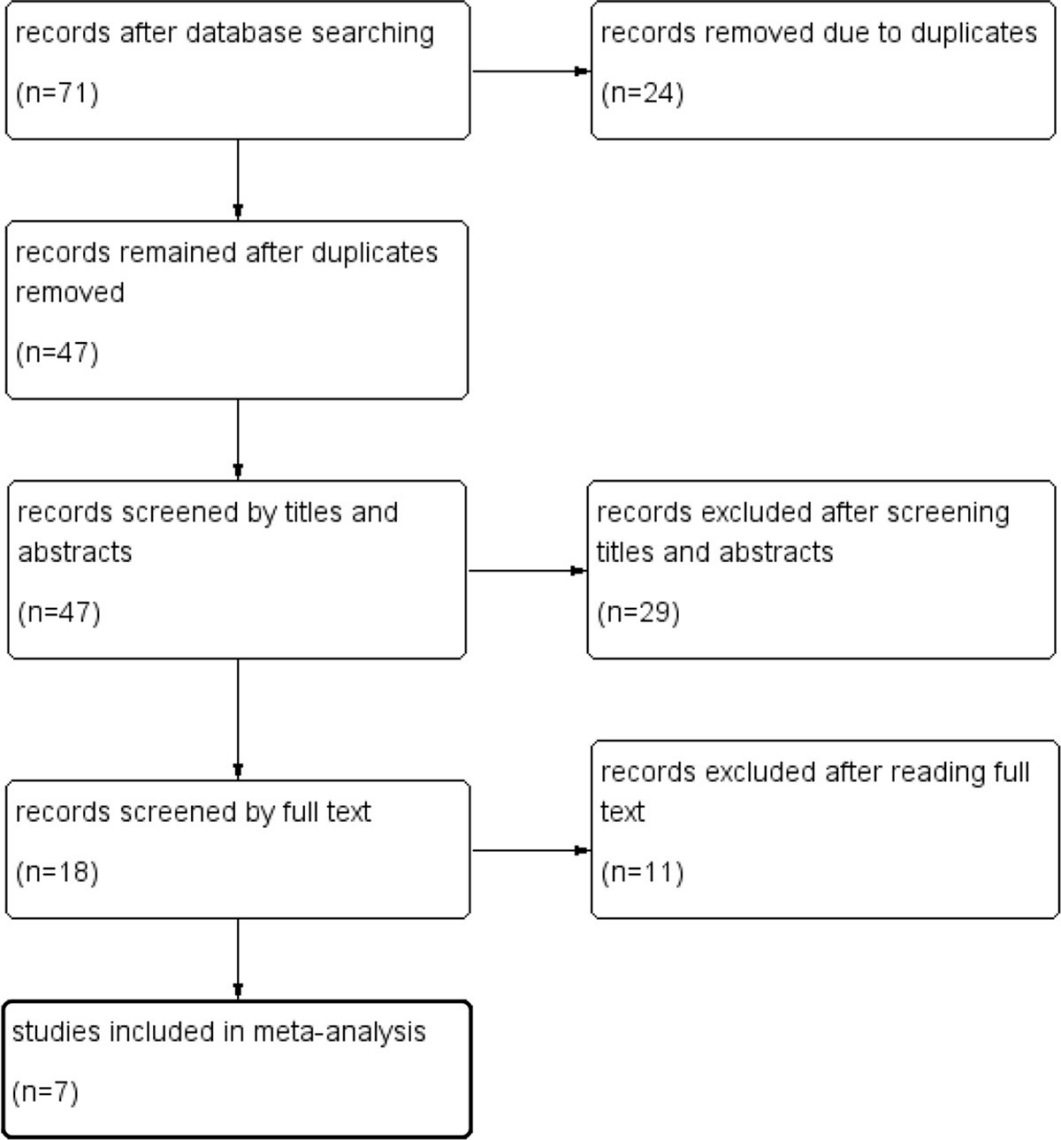
The process of study selection.

The search strategy resulted in 71 references; -after removing 24 duplicate articles, 47 articles remained. We excluded 29 articles that were obviously irrelevant based on the titles and abstracts, and the full text of the remaining 18 was assessed in detail. After full-text assessment, we eventually identified 7 studies [7, 10–15] that could be used for the meta-analysis. A flowchart of the process used for selection of the studies is presented in Fig 1.

### Study characteristics

Our meta-analysis included 7 studies that examined the association of related factors with SB in PD; a total of 1354 PD patients were enrolled in these studies. The 7 studies were performed in 5 countries around the word. One study was conducted in China, Australia, Norway, respectively, and three of the studies were conducted in Holland. There was a median of 193 PD patients per study. The characteristics of these 7 studies are shown in Table 1. All the included studies had a quality score over 6 according to the Newcastle -Ottawa scales, indicating good quality.

**Table 1:** The characteristics of the included studies.

Publication	Publication date	Country	Race	Diagnostic criteria	N	SB	NSB
Du et al.	2018	China	Chinese	UKPDSBB	100	51	49
W.Lee et al.	2017	Australia	Caucasian	UKPDSBB	92	20	72
Van Gilst et al.	2015	Holland	Caucasian	Not reported	237	74	163
E.Sherif et al.	2014	Holland	Caucasian	Not reported	131	39	92
M.M.van Gilst et al.	2012	Holland	Caucasian	UKPDSBB	243	114	129
E.Tandberg et al.	1999	Norway	Caucasian	Not reported	239	101	138
M.Merello et al.	1996	Germany	Caucasian	UKPDSBB	312	172	140

N, number; SB, sleep benefit group; BSB, no sleep benefit group; UKPDSBB: the clinical criteria for PD according to the United Kingdom Parkinson’s Disease Society Brain Bank of London.

### Meta-analysis

The following possible related factors of SB in patients with PD were analyzed by pooling the results in our meta-analysis: gender, age, age at PD onset, time of PD from diagnosis, time on treatment, LEDD (L-dopa equivalent daily dose), time to fall asleep, PSQI total score (Pittsburg Sleep Quality Scale), total sleep duration, sleep efficiency, ESS total score (Epworth Sleepiness Scale total score), on-state MDS-UPDRS-Ⅲ total score, and Hoehn-Yahr scale.

**Fig 2:**
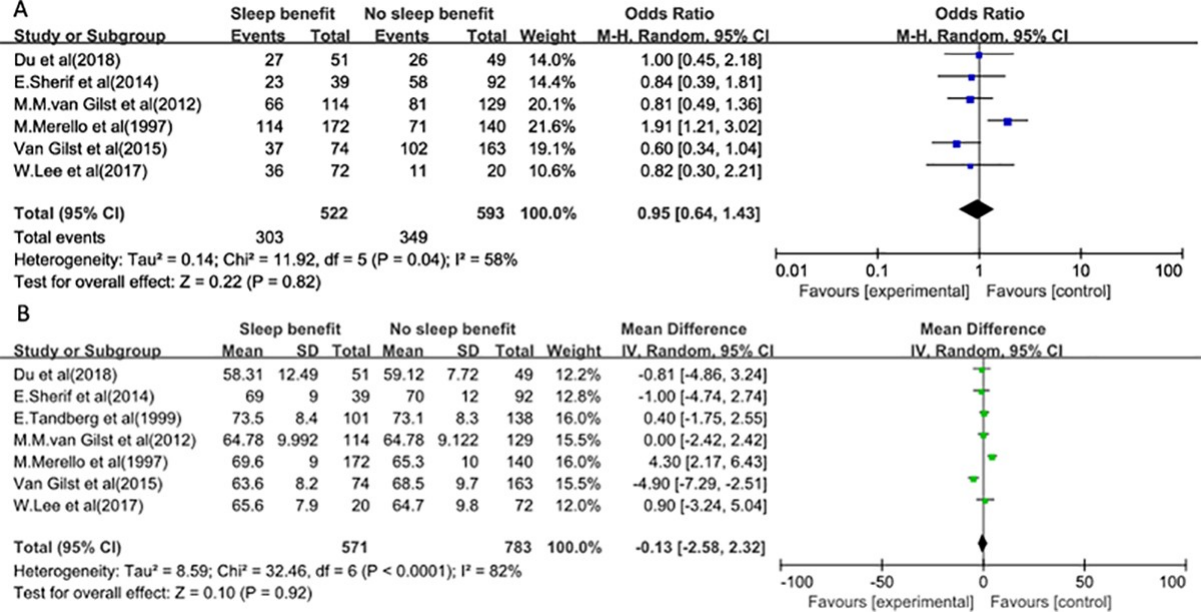
Meta-analysis of the associations of the related basic factors with SB. (A) Gender. (B) Age.

**Fig 3:**
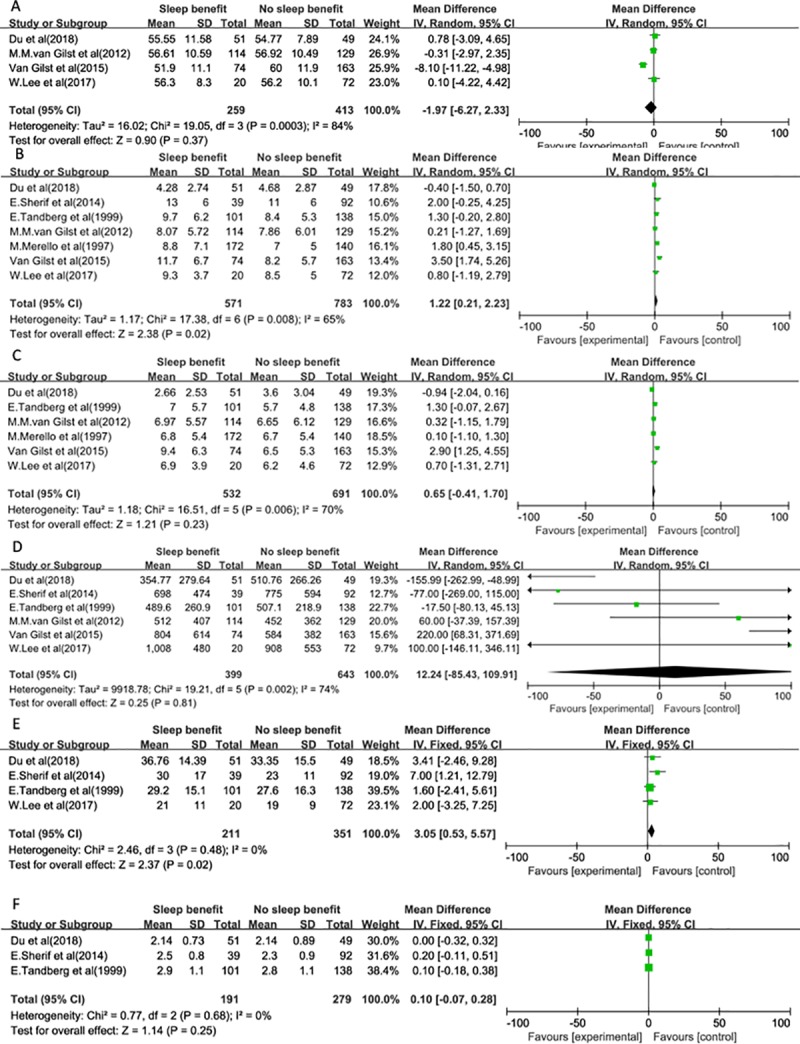
Meta-analysis of the associations of related PD factors with SB. (A) Age at PD onset. (B) Time of PD from diagnosis. (C) Time on treatment. (D) LEDD. (E) On-state MDS-UPDRS-Ⅲ total score. (F) Hoehn-Yahr scale.

**Fig 4:**
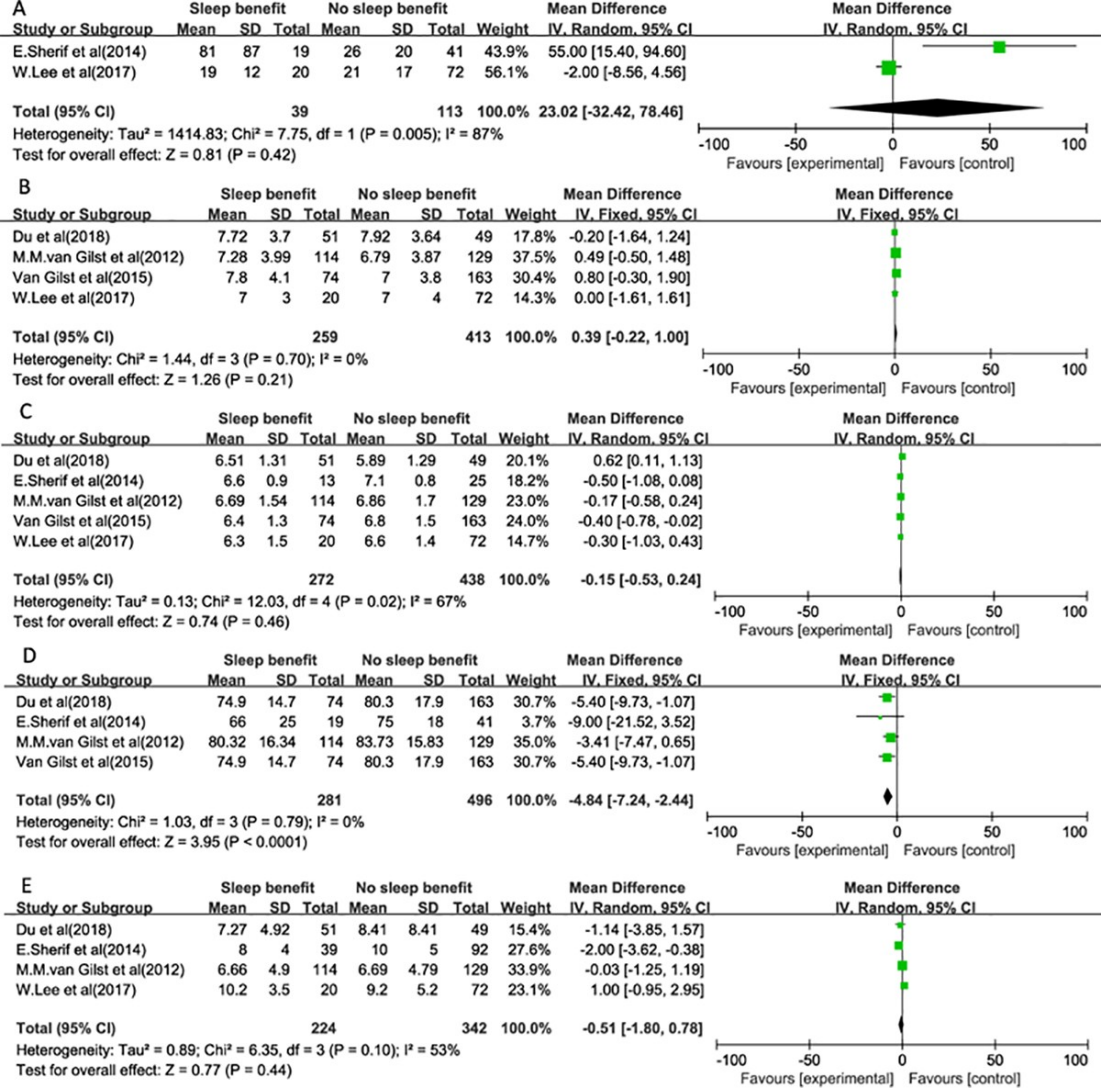
Meta-analysis of the associations of related sleep factors with SB. (A) Time to fall asleep. (B) PSQI total score. (C) Total sleep duration. (D) Sleep efficiency. (E) ESS total score.

A total of 13 possible related factors were analyzed. We further determined the associations of these possible factors with SB in PD. No statistically significant differences were observed between SB in PD and NSB in PD in our meta-analysis for the following factor: gender (OR 0.95, 95% CI: 0.64–1.43),age (MD -0.13, 95% CI: -2.58–2.32), age at PD onset (MD -1.97, 95% CI: -6.27–2.33), time on treatment (MD 0.65, 95% CI: -0.41–1.70), LEDD (MD 12.24, 95% CI: -85.43–109.91), time to fall asleep (MD 23.02,95% CI: -32.42–78.46), PSQI total score (MD 0.39,95% CI: -0.22–1.00), total sleep duration (MD -0.15,95% CI: -0.53–0.24), ESS total score (MD -0.51,95% CI: -1.80–0.78), Hoehn-Yahr scale (MD 0.10,95% CI: -0.07–0.28). We found 3 factors associated with SB. First, 7 studies were included to assess the association of the duration of PD with SB, and we observed a significant association between the duration of PD and SB in PD (MD 1.22, 95% CI: 0.21–2.23). Second, there was a significant difference between SB and NSB for sleep efficiency by polling the results from the fixed- effects model (MD-4.48,95% CI: -7.24- -2.44). Third, a significant association was found between the on-state MDS-UPDRS-Ⅲ total score and SB in PD patients (MD 3.05,95% CI: 0.53–5.57). The forest plots are presented in Figs 2–4.

The heterogeneity tests were significant for gender (Q test: p = 0.04 and I^2 test = 58%), age (Q test: p <0.0001 and I^2 test = 82%), age at PD onset (Q test: p = 0.0003 and I^2 test = 84%), time of PD from diagnosis (Q test: p = 0.008 and I^2 test = 65%), time of treatment (Q test: p = 0.006 and I^2 test = 70%), LEDD (Q test: p = 0.002 and I^2 test = 74%), time to fall asleep (Q test: p = 0.005 and I^2 test = 87%), total sleep duration (Q test: p = 0.02 and I^2 test = 67%), and ESS total score (Q test: p = 0.10 and I^2 test = 53%). In these cases, the estimations were based on random effects models.

## Discussion

This systematic review and meta-analysis included 7 clinical observational studies that assessed the associations of related risk factors with SB in PD patients. We showed that PD duration, sleep efficiency and on-state MDS-UPDRS-Ⅲ total score were significantly associated with SB in PD. In other words, PD patients with SB are more likely to have a long duration of PD, a low sleep efficiency and a high MDS-UPDRS-Ⅲ total score.

The prevalence of SB is consistently reported to be quite high, with 33%-55% of PD patients reporting the experience of SB[7, 16]. Conversely, half of PD patients do not report SB, which remains interesting. However, its underlying mechanisms remain unclear, and several hypotheses have been proposed. One leading hypothesis on the mechanism of sleep benefit stated that dopamine storage in nigral neuronal terminals are replenished during sleep[8, 17], and this would not fit with the relationship between low sleep quality and SB in our meta-analysis; thus, this finding requires further study. However, that hypothesis was questioned by the study of Van Gilst et al[18]. That study suggested that SB is an overnight medication effect, caused by extended release of L-dopa or longer acting dopamine agonists. This finding was highlighted by a trend toward more daily L-dopa equivalent medication use in the SB group in their study. However, in our meta-analysis, no significant difference was found for LEDD between the SB group and the NSB group, indicating that the conclusion of the study by Van Gilst et al seems less likely. A significant difference in the medication response between SB and NSB was found: following a levodopa-induced “on” period, patients with sleep benefit had a more severe inter-dose”off” than those without SB[17]. S. A. Factor et al. [19] proposed another hypothesis that SB actually represented a “morning benefit” and is a pattern of motor fluctuations likely, unrelated to sleep. These motor fluctuations commonly occur in patients who have used levodopa over a long period of time. However, our meta-analysis did not determine that SB was associated with the duration of drug treatment. Furthermore, the occurrence of SB after daytime naps suggest a specific role of sleep[14]. Moreover, sleep efficiency was associated with SB in PD patients in our meta-analysis. Patient without motor fluctuations can also experience SB, and many patients without SB have motor fluctuations. This evidence dose not support the hypothesis of S. A. Factor et al. Another hypothesis was raised that patients with sleep deprivation were more likely to experience SB[17], which was confirmed by our finding that low sleep efficiency was associated with SB. One hypothesis is that SB might be explained by physiological circadian fluctuations in dopamine levels. However, B. H. Hogl et al. explored the impact of circadian rhythms on SB and did not find a significant difference in the type of circadian synchronization[17]. Previous studies found that patients with SB used higher doses of dopa minergics[20]. The higher dose of medicine may be associated with SB, but our meta-analysis did not duplicate this finding. The significant variation may cause the different results.

To the best of our knowledge, our meta-analysis is the first work on this topic. We identified 3 related risk factors associated with SB in PD: PD duration, sleep efficiency and on-state MDS-UPDRS-Ⅲ total score. Our work helps obtain a better understanding of SB and its underlying mechanisms. Very few studies were included in this meta-analysis. Therefore, our study highlights the need for conducting other studies on these risk factors using valid methodologies. This meta-analysis has several limitations. First, a meta analysis may be biased when the literature search fails to identify all relevant studies. However, access to unpublished articles and insufficient data in published studies remains difficult. To minimize these risks, we performed thorough searches across multiple literature databases. Furthermore, we did not investigate publication bias due to the limited number of included studies. Second, it is important to realize that the considerable variation in the outcomes of studies assessing possible risk factors of SB can in part be explained by the application of different definitions of SB. The heterogeneity identified in this meta-analysis might be partly explained by the difference in SB definitions (subjective-reporting or objective measurements). The different measurements of these factors may also lead to heterogeneity. Therefore, we used the random effects model to pool the results for significant heterogeneity in our meta-analysis. Third, several factors can not be analyzed for various reasons, such as depression due to the use of different questionnaires in the included studies. In addition, we only reported LEDD in our meta-analysis, and we did not take long-acting drugs into consideration.

In conclusion, this work provided us with knowledge on the related factors associated with SB in PD patients. We identified 3 factors related to SB: PD duration, sleep efficiency and on-state MDS-UPDRS-Ⅲ total score. Our work helps obtain a better understanding of SB and its underlying mechanisms.

SB remains a fascinating, but mysterious phenomenon. These is a need to conduct more studies to evaluate the associations of clinical characteristics in PD with SB phenomenon. For this purpose, the methodology of the studies, the definitions of SB and the measurements of these factors should be standardized. In addition, further study should assess the underlying mechanisms of SB.

## Supporting information

S1 File MOOSE checklist. (DOC)

S2 File PRISMA checklist. (PDF)
