# Correction: The related factors of sleep benefit in Parkinson's disease: A systematic review and meta-analysis

**DOI:** 10.1371/journal.pone.0216524

**Published:** 2019-05-01

**Authors:** Rui Zhong, Qingling Chen, Xinyue Zhang, Xin Zhang, Weihong Lin

The first and last names of all of the authors are incorrectly switched. The correct names are: Rui Zhong, Qingling Chen, Xinyue Zhang, Xin Zhang, Weihong Lin. The correct citation is: Zhong R, Chen Q, Zhang X, Zhang X, Lin W (2019) The related factors of sleep benefit in Parkinson’s disease: A systematic review and meta-analysis. PLoS ONE 14(3): e0212951. https://doi.org/10.1371/journal.pone.0212951
