# Supplementary material for: The related factors of sleep benefit in Parkinson’s disease: A systematic review and meta-analysis
Source: PLoS One. 2019 Mar 11;14(3):e0212951. doi: 10.1371/journal.pone.0212951 (PMC6411108; doi:10.1371/journal.pone.0212951)
Supplement: S1 File — (DOC) [file pone.0212951.s001.doc]

**MOOSE Checklist**

### The related factors of sleep benefit in Parkinson’s disease: A systematic review and meta-analysis

| **Criteria** | | **Page** |
| --- | --- | --- |
| **Reporting of background should include** | | |
| 1 | Problem definition | 2 |
| 2 | Hypothesis statement | 2 |
| 3 | Description of study outcomes | 2 |
| 4 | Type of exposure or intervention used | 3 |
| 5 | Type of study designs used | 3 |
| 6 | Study population | 3 |
| **Reporting of search strategy should include** | | |
| **7** | Qualifications of searchers | 3 |
| 8 | Search strategy, including time period included in the synthesis and keywords | 3 |
| 9 | Databases and registries searched | 3 |
| 10 | Search software used, name and version, including special features | 3 |
| 11 | Use of hand searching | 3 |
| 12 | List of citations located and those excluded, including justifications | 3 |
| 13 | Method of addressing articles published in languages other than English | 3 |
| 14 | Method of handling abstracts and unpublished studies | 3 |
| 15 | Description of any contact with authors | NONE |
| **Reporting of methods should include** | | |
| 16 | Description of relevance or appropriateness of studies assembled for assessing the hypothesis to be tested | 4 |
| 17 | Rationale for the selection and coding of data | 4 |
| 18 | Assessment of confounding |  |
| 19 | Assessment of study quality, including blinding of quality assessors; stratification or regression on possible predictors of study results | 3 |
| 20 | Assessment of heterogeneity | 4 |
| 21 | Description of statistical methods in sufficient detail to be replicated | 4 |
| 22 | Provision of appropriate tables and graphics | 4 |
| **Reporting of results should include** | | |
| 23 | Graph summarizing individual study estimates and overall estimate | 5 |
| 24 | Table giving descriptive information for each study included | 5 |
| 25 | Results of sensitivity testing | NONE |
| 26 | Indication of statistical uncertainty of findings | 6 |
| **Reporting of discussion should include** | | |
| 27 | Quantitative assessment of bias | NONE |
| 28 | Justification for exclusion | 6 |
| 29 | Assessment of quality of included studies | 6 |
| **Reporting of conclusions should include** | | |
| 30 | Consideration of alternative explanations for observed results | 7 |
| 31 | Generalization of the conclusions | 7 |
| 32 | Guidelines for future research | 7 |
| 33 | Disclosure of funding source | NONE |
